# Early detection of diabetic retinopathy based on deep learning and ultra-wide-field fundus images

**DOI:** 10.1038/s41598-021-81539-3

**Published:** 2021-01-21

**Authors:** Kangrok Oh, Hae Min Kang, Dawoon Leem, Hyungyu Lee, Kyoung Yul Seo, Sangchul Yoon

**Affiliations:** 1 Research Institute of Radiological Science, College of Medicine, Yonsei University, Seoul, 03722 Republic of Korea; 2 Department of Ophthalmology, College of Medicine, Catholic Kwandong University, International St. Mary’s Hospital, Incheon, 22711 Republic of Korea; 3 DoAI Inc., Seoul, 06148 Republic of Korea; 4 Department of Ophthalmology, College of Medicine, Yonsei University, Seoul, 03722 Republic of Korea; 5 Department of Medical Humanities and Social Sciences, College of Medicine, Yonsei University, Seoul, 03722 Republic of Korea

**Keywords:** Retinal diseases, Biomedical engineering

## Abstract

Visually impaired and blind people due to diabetic retinopathy were 2.6 million in 2015 and estimated to be 3.2 million in 2020 globally. Though the incidence of diabetic retinopathy is expected to decrease for high-income countries, detection and treatment of it in the early stages are crucial for low-income and middle-income countries. Due to the recent advancement of deep learning technologies, researchers showed that automated screening and grading of diabetic retinopathy are efficient in saving time and workforce. However, most automatic systems utilize conventional fundus photography, despite ultra-wide-field fundus photography provides up to 82% of the retinal surface. In this study, we present a diabetic retinopathy detection system based on ultra-wide-field fundus photography and deep learning. In experiments, we show that the use of early treatment diabetic retinopathy study 7-standard field image extracted from ultra-wide-field fundus photography outperforms that of the optic disc and macula centered image in a statistical sense.

## Introduction

Diabetic retinopathy (DR) is responsible for 0.8 million blind and 3.7 million visually impaired people globally in 2010^1^. Due to the increasing number of diabetes patients, the number of DR patients has been estimated to be 191.0 million by 2030^2,3^. Though the global prevalence of any DR was 27.0% for the period 2015 to 2019^4^, there are no distinct symptoms at the early stages of DR, including the referable DR. Since DR can be fairly advanced before affecting vision^2^, timely diagnosis and treatment can reduce the risk of visual loss by approximately 57%^5^. Therefore, routine screening and regular follow-up are essential for patients with diabetes, especially middle age and aged people. However, several studies^6–8^ have indicated that a significant amount of patients with diabetes failed to have recommended annual eye examination due to long examination time, lack of symptoms, and limited access to retinal specialists.

One of the efforts to resolve these barriers is the application of artificial intelligence (AI)^9^ techniques for DR detection and diagnosis. In 2016, Gulshan et al.^10^ developed a deep learning (DL) algorithm for DR evaluation. In the study, they trained their model using approximately 0.13 million training images. As a result, area under the receiver operating characteristic curve (AUC) values of 0.97–0.99 were obtained from tests using two separate data sets for detecting referable DR. Abramoff et al.^11^ developed an automated system using convolutional neural networks (CNNs) for DR detection on a publicly available dataset. Since these pioneering studies, several research works focused on adopting DL technology for DR detection^12^ and grading^13,14^. Furthermore, Gulshan et al.^15^ prospectively validated the performance of a DR grading system comparing to that of manual grading across two sites in India. A deep learning system (DLS) considering glaucoma and age-related macular degeneration (AMD), as well as DR, was studied for multiethnic populations with diabetes by Ting et al.^16^. These representative studies utilized conventional fundus photography, which captures the optic nerve and macula with a field of view (FOV) between $20^\circ$ and $50^\circ$. Though conventional fundus photography contains the most crucial region for DR detection and diagnosis, there is a large portion of the uncaptured retinal surface.

Takahashi et al.^17^ utilized non-mydriatic $45^\circ$ fundus photographs of four-field to capture a wide retinal area for DR staging based on a DL algorithm. In the study, the use of four-field fundus photography showed better grading performance than a single field fundus photography for DR grading. However, the acquisition of four-field fundus photography can be time-consuming and require considerable effort. With the advancement in retinal imaging technology, ultra-wide-field (UWF) fundus photography provides $200^\circ$ of retinal surface images in a single shot^18^, providing both posterior pole and peripheral retinal images. UWF retinal images including UWF fluorescein angiography are now widely accepted for DR diagnosis and treatment, providing peripheral neovascularization and ischemic areas^19^. Nagasawa et al.^20^ investigated proliferative diabetic retinopathy (PDR) detection based on UWF fundus photography and deep learning algorithm. In the study, they acquired high sensitivity, specificity, and AUC on a relatively small in-house dataset. To our knowledge, the automated DR detection and grading system based on deep learning technology is not investigated thoroughly. In this study, we present the development and validation of a DLS for DR detection based on UWF fundus photography collected during routine DR evaluation from clinical settings in a hospital located in South Korea. Our study is a feasibility study based on single-center, single-ethnicity, and single-device data.

The purpose of our study is to investigate the effectiveness of UWF fundus photography in DR detection. However, the UWF fundus photography contains artifacts such as periocular regions placed mostly outside the early treatment of diabetic retinopathy study (ETDRS) 7-standard field (7SF). Besides, ETDRS 7SF is the most prevalent region for DR detection and diagnosis tasks. For these reasons, we limit the region of interest (ROI) to the ETDRS 7SF for the DR detection task based on UWF fundus photography. In this study, we develop and investigate a DR detection system based on ETDRS 7SF, which is the most significant region of UWF fundus photography. Furthermore, we segment ETDRS Field 1 and Field 2 (F1–F2) regions for comparison purposes. We note here that the ETDRS F1–F2 image is a reasonable alternative to the standard fundus image.

## Methods

**Figure 1:**
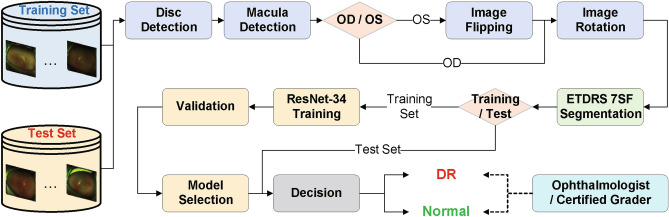
An overview of the proposed DR detection system.

The proposed DR detection system requires an automatic segmentation of the ETDRS 7SF to remove undesirable components such as eyelashes and skin. Using the segmented ROI image, we employ the deep learning architecture, the residual network with 34-layer (ResNet-34) model^21^ as a classifier for the DR detection task. Figure 1 shows an overview of the proposed DR detection system. To evaluate the DR detection performance, we compare our system with the one based on the ROI containing only the ETDRS Field 1 and Field 2 (F1–F2) in terms of several metrics. We note here that the ETDRS F1–F2 image is an alternative of the conventional single or two non-mydriatic 45-degree fundus photography under a condition that the UWF and conventional fundus images are not coexisting.

### Ultra-wide-field fundus photography acquisition

For evaluation of the system performance, we have acquired a set of UWF fundus images from the Catholic Kwandong University International St. Mary’s Hospital, South Korea. Catholic Kwandong University International St. Mary’s Hospital institutional board reviewed and approved this study (IS19RISI0005). All research was performed in accordance with relevant guidelines and regulations. Informed consent was obtained from all subjects or if subjects are under 18, from a patient and/or legal guardian. We also note that informed consent was obtained from the participants/patients to publish the information/images in an online open access publication. The capturing device for the UWF fundus photography is Optos Daytona UWF retinal imaging system. The obtained image size is $3072 \times 3900$ pixels. The in-house dataset consists of 11,734 and 1537 UWF fundus photographs of DR patients and healthy subjects. The total number of patients is 1308, where their ages are ranging from 8 to 89, and the average age is 50.76. The percentile of patients whose ages are between 40 and 70 is $72.1\%$. The proportion of males to the total patients is $50.75\%$. An ophthalmologist and a certified grader have participated in grading based on the ETDRS protocols. The acquired in-house dataset is a binary class (healthy and DR) data of which DR severity level is in the moderate and severe non-proliferative diabetic retinopathy (NPDR) stages. The ophthalmologist with more than ten years of experience and the certified grader with two years of experience have performed the grading independently. Furthermore, they have checked an image twice with concealing the previously made grading outcomes. We exploit the UWF fundus images with concurrent intra-observer and inter-observer grading outcomes for our experiments.

### Optic disc and macula detection

**Figure 2:**
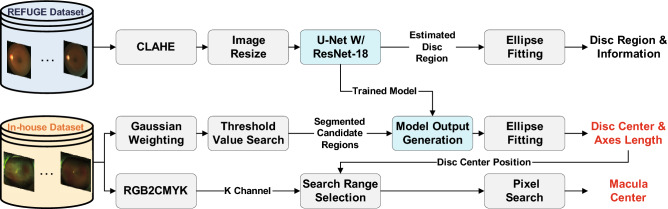
The overall flow of the optic disc and macular detection process.

UWF fundus images may contain periocular regions such as eyelids and eyelashes that are undesirable for DR detection. To exclude these regions in DR detection, we extract the ROI based on the optic disc and macula centers. Figure 2 illustrates the optic disc and macula detection process. In our system, optic disc and macula center positions are the reference points for UWF fundus photography alignment. Our system utilizes the U-Net model^22^ with the pre-trained residual network with 18-layer (ResNet-18) model^21^ as the encoder for optic disc detection. The ImageNet dataset^23^ is utilized for the pre-training. To train the U-Net model, we employ the publicly available Refuge dataset. As image pre-processing, contrast-limited adaptive histogram equalization (CLAHE)^24^ and bicubic interpolation^25^ based image resize are adopted. Subsequently, we train and test the U-Net model using the processed images with a size of $512 \times 512$ to estimate the optic disc region. Consequently, the optic disc center position and axes length is obtained by employing an ellipse fitting methodology. We utilize the trained U-Net model for estimating the optic disc region in UWF fundus images.

**Figure 3:**
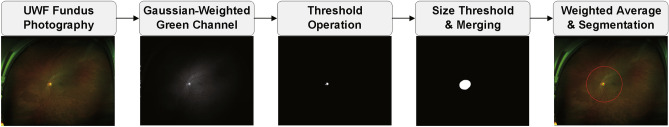
Sample images at each processing stages.

Since the UWF fundus images in our in-house dataset have a relatively bigger size and capture different areas comparing to standard fundus images, our system searches for image sub-regions before applying the trained U-Net model. Firstly, pixel-wise Gaussian weighting is applied to the green channel of the original UWF fundus images to exclude pixels with large intensity values near image boundaries (e.g., skin). Using the Gaussian-weighted images, threshold operation is performed based on pixel intensity. For the threshold value, we calculate mean ($\mu$) and standard deviation ($\sigma$) values from the optic disc regions of 50 UWF fundus images. The threshold $\tau$ is set as $\tau = \mu - \sigma$. After the threshold operation, there can be multiple detected areas that are candidates of the optic disc region. To exclude the erroneously detected regions, we apply a size threshold operation and merge adjacent regions within 150 pixels. Additionally, from the circular region around each center of the remaining candidates, the center position of pixels with intensity values larger than 50 in the red channel of the original UWF fundus image is detected. The weighted average of the pixel positions from the red and green channels (0.75 for the green channel and 0.25 for the red channel) is calculated and utilized as the centers of candidates for the optic disc region. Eventually, we segment $614 \times 614$ circular area focused at each center of the optic disc candidates and resize the image to $512 \times 512$. Figure 3 shows sample images from the aforementioned processing stages.

The model outputs of the segmented candidate images are generated by the U-Net model trained using the Refuge dataset. For optimization, we utilize Adam optimizer^26^ with a learning rate of 0.0001. The number of epochs is set to 30, and dice loss is utilized. Among the multiple segmented candidate images, we choose the one with the highest model outputs. As a result, the system estimates pixels that belong to the optic disc region. Subsequently, the optic disc center position and its axes length are extracted based on an ellipse fitting for further processing. To detect the macula center, the system transforms the color space of the image from RGB to CMYK. Subsequently, the system searches for a pixel with the smallest intensity in the K channel. The search range is defined as a rectangular region that is 500 pixels horizontally and 30 vertically away from the optic disc center. We define the detected pixel as the macula center in our system. The detected optic disc and macula centers are the reference points for further image alignment. We note that we obtained successful optic disc detection results for 10,442 images from DR patients and 1442 images from normal subjects among 13,271 UWF fundus images in the in-house dataset.

**Figure 4:**
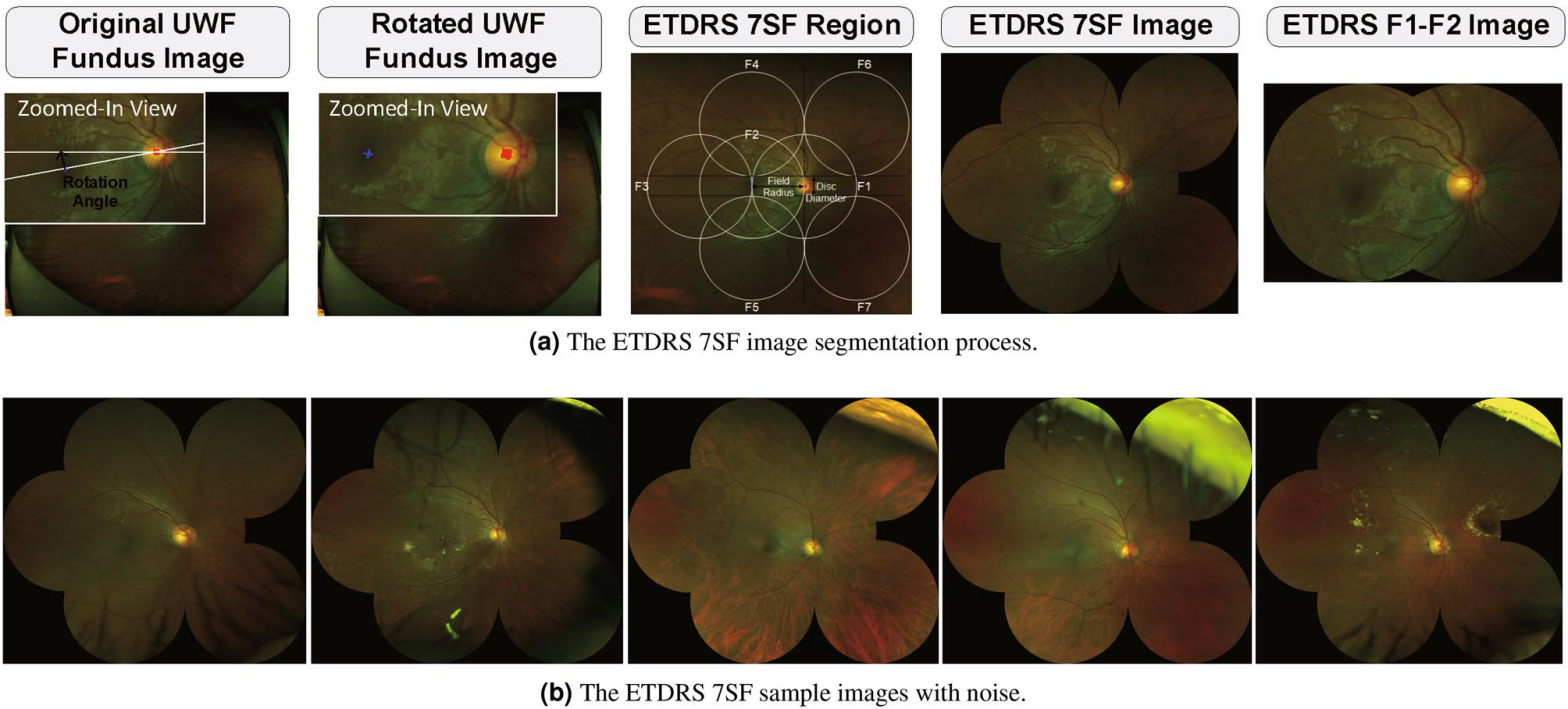
The ETDRS 7SF image segmentation process and sample images with noise.

### ETDRS 7 standard fields image segmentation

As an image alignment, we segment the ETDRS 7SF image from the original UWF fundus photography using the optic disc and macula centers. For convenience at the training phase, the system transforms OS images to OD-like ones based on image flipping in the horizontal direction. As a consequence, the optic disc region is always on the right side of the macula. Since the optic disc and macula centers are located at different row indices of an image, it is necessary to rotate the image to arrange those centers evenly. Hence, we rotate the image around the optic disc center. From the rotated image, we segment the ETDRS 7SF based on the optic disc and macula centers. The segmented ETDRS 7SF and F1–F2 images are resized to the size of $896 \times 1024$ and $448 \times 640$, respectively. Figure 4a illustrates the ETDRS 7SF image segmentation process and Fig. 4b shows images with unwanted components such as eyelashes and eyelids. We note here that the segmented images with these components are excluded in the evaluation process. Finally, we obtained successful ETDRS 7SF segmentation results for 7282 images from DR patients and 1101 images from normal subjects.

### ResNet-34 model training

**Figure 5:**
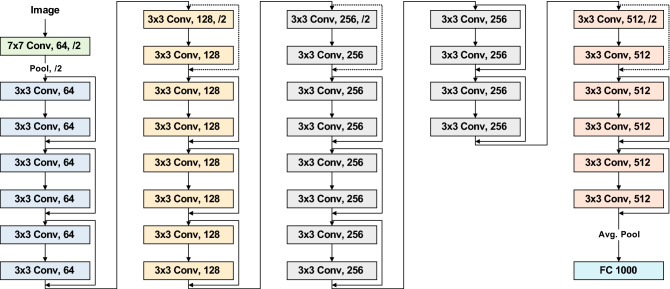
The ResNet-34 model.

Our DR detection system utilizes the ResNet-34 model^21^ for the classification task since our dataset is relatively small and it is binary class data. The ResNet-34 model utilized in our system is pre-trained on ImageNet^23^, and finetuned on the in-house dataset. Figure 5 illustrates the ResNet-34 model. The ResNet architecture provides advantages in an easier optimization and accuracy gain for deep networks^21^. To handle data imbalance between classes, we utilize weighted loss based on the number of training samples in the minority class (*N*). Weight for each class is obtained by dividing *N* by the number of training samples in each class. For optimization, the stochastic gradient descent with 0.001 learning rate and 0.9 momenta is utilized while the learning rate is set to decay by a factor of 0.1 for every 7 epochs. The number of epochs is set at 25.

## Results

### Evaluation protocols

In our experiments, automated DR detection systems using the two segmentation images are assessed. To set the single field size identical between two types of images, the ETDRS 7SF and F1–F2 images are normalized to $896\times 1024$ and $448\times 640$ pixels respectively. For detection system performance evaluation, ten runs of ten-fold stratified cross-validation tests are performed using the acquired in-house UWF fundus images dataset since there is no publicly available data. At the validation phase, a single run of ten-fold hold-out validation is performed using the training set only. Consequently, $90\%$ and $10\%$ of images in the training set are utilized for training and validation tasks, respectively.

As for performance indicators, we employ the accuracy, AUC, sensitivity, and specificity, where average and standard deviation values are reported for each metric. We note that the operating threshold value for sensitivity, specificity and accuracy measures is set when the sensitivity and specificity performances are the most similar. Additionally, a paired-sample *t* test is performed to verify whether the performance gap between systems based on ETDRS 7SF and F1–F2 images is meaningful in a statistical sense. For analysis purpose, we also visualize the class activation maps (CAM) to indicate the discriminative image regions which contribute to decision making according to a technique based on the global average pooling layer^27^. Furthermore, we report the repeatability of the test model outputs regarding the relative standard deviation (RSD)^28^. We measure image-wise RSD values using the test model outputs from the ten runs of cross-validation tests. Consequently, average RSD values for both DR detection systems based on ETDRS 7SF and F1–F2 images are reported.

### Detection performance assessment

**Figure 6:**
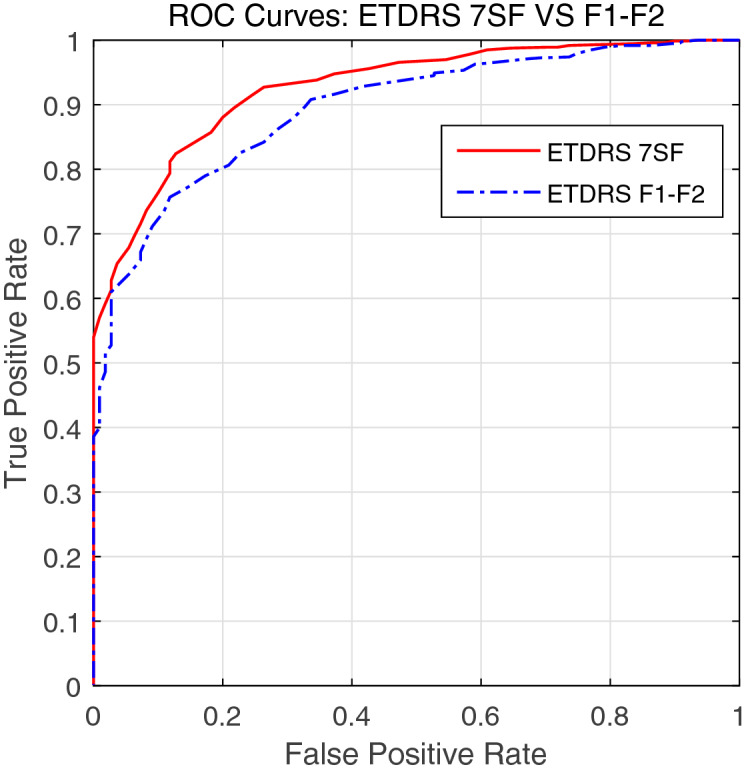
The ROC curves of the DR detection system using ETDRS 7SF and F1–F2 fundus images. Here, we note that the true positive rate and the false positive rate for plotting are obtained from a single running fold experiment among the entire cross-validation tests.

**Figure 7:**
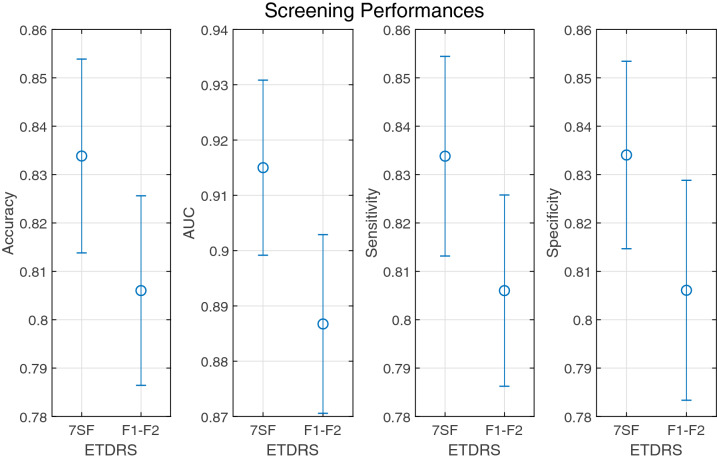
The DR detection performances of the system using ETDRS 7SF and F1–F2 fundus images in terms of accuracy, AUC, sensitivity, and specificity. For each metric, plots show the mean (marked with blue circle) and deviations (marked with blue bar).

To provide a comprehensive detection performance throughout the overall range of decision thresholds, the ROC curves are plotted in Fig. 6. Across the entire range, the DR detection system based on ETDRS 7SF images outperform that based on ETDRS F1–F2 due to the exploitation of supplementary information at the peripheral region which is not visible in ETDRS F1–F2 images. Figure 7 shows the mean and deviations of accuracy, AUC, sensitivity, and specificity which are acquired from ten runs of ten-fold stratified cross-validation tests. In terms of all metrics, the DR detection system using ETDRS 7SF images tends to perform better than that using ETDRS F1–F2 images.

**Figure 8:**
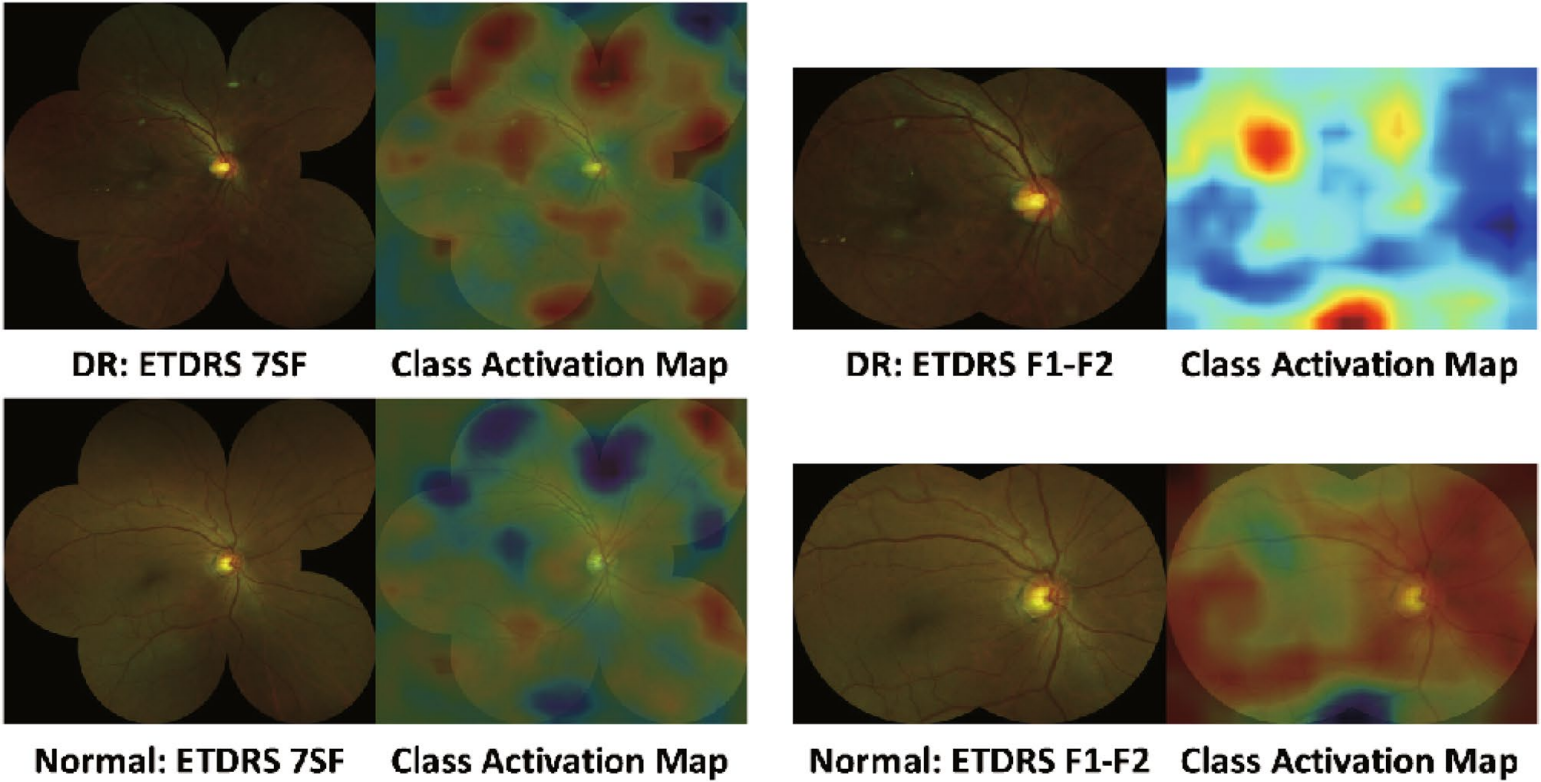
Class activation maps generated from ETDRS 7SF and F1–F2 images for DR and normal class.

The mean and standard deviation values of accuracy, AUC, sensitivity, and specificity metrics are provided in Table 1. Both systems perform tolerably in terms of accuracy, AUC, and sensitivity. For specificity, the DR detection system based on ETDRS 7SF images performs significantly better than that based on ETDRS F1–F2 images. This means that the regions outside ETDRS F1–F2 provide supplementary information that is useful for discrimination between DR and normal class. To support this, we present CAM images acquired by the systems using ETDRS 7SF and F1–F2 images in Fig. 8.

**Table 1:** The sensitivity, specificity, accuracy, and AUC results of the DR detection system using ETDRS 7SF and F1–F2 fundus images. The values are represented as the form of $\mu \pm \sigma$, where $\mu$ and $\sigma$ denote the mean and standard deviation, respectively.

Performance metric	Accuracy	AUC	Sensitivity	Specificity
ETDRS 7SF	$0.8338\pm 0.0047$	$0.9150\pm 0.0048$	$0.8338\pm 0.0048$	$0.8341\pm 0.0042$
ETDRS F1–F2	$0.8060\pm 0.0054$	$0.8867\pm 0.0037$	$0.8060\pm 0.0053$	$0.8061\pm 0.0069$

**Table 2:** Results from the paired-sample *t* test. The significance level ($\alpha$) is set at $\alpha =0.001$.

Performance metric	Accuracy	AUC	Sensitivity	Specificity
Probability Value (*p*)	${p<0.0001}$	${p<0.0001}$	${p<0.0001}$	${p<0.0001}$
Hypothesis Test Result (*H*)	1	1	1	1

### Statistical significance tests

To verify whether the performance gap between the DR detection systems based on the ETDRS 7SF and F1–F2 images is meaningful in a statistical sense, we adopt a paired-sample *t* test^29^ using test sensitivity, specificity, accuracy, and AUC measures from ten runs of ten-fold stratified cross-validation tests. From the paired-sample *t* test, we investigate the impact of including peripheral regions outside the F1–F2 in the DR detection process. The test outcome $H=0$ stands for retaining a null hypothesis that the DR detection based on the ETDRS 7SF and F1–F2 images perform equivalently. On the other hand, $H=1$ means that performance enhancement by the inclusion of peripheral regions in the DR detection process is statistically significant. The confidence level $\alpha$ is set at $\alpha =0.001$. Table 2 shows results from the paired-sample *t* test using the four performance metrics. As shown in the table, the performance enhancement for all metrics is statistically significant. From the repeatability test, average RSD values of $12.85\%$ and $15.10\%$ are reported for DR detection systems based on the ETDRS 7SF and F1–F2 images, respectively. The DR detection system based on the ETDRS 7SF images shows relatively more precise results than the system based on the ETDRS F1–F2 images.

## Discussion

Conventional fundus cameras capture the optic nerve and macula with a FOV between $20^\circ$ and $50^\circ$^30^. Despite the resulting single-field fundus photography contains the most significant area, a large portion of the retina is still not captured. The ETDRS 7SF photography^5^ was developed by combining $30^\circ$ field images to resolve the limitation. It captures approximately $90^\circ$ of the retina that is around $30\%$ of the retinal surface^31^. Since 1991, the ETDRS 7SF photography has been the gold standard for the classification and severity evaluation of DR^32^. However, acquisition of the ETDRS 7SF images is not as convenient as that of the single-field fundus images since it requires skilled photographers and is time-consuming^30^.

With the recent advancement of the high-resolution UWF imaging, up to $82\%$ of the retinal surface can be captured in a single image^33^. Several study groups obtained a high level of agreement from a comparison between the UWF photography and the ETDRS 7SF photography for DR evaluation^33–36^. Furthermore, Silva et al. demonstrated that peripheral lesions identified on UWF imaging are associated with the increased risk of DR progression^37^. Those pioneering studies^33–37^ regarding the UWF imaging for DR severity evaluation utilized capturing devices from Optos. The wide-field scanning laser ophthalmoscopy (SLO) by Optos provides a single image covering nearly $200^\circ$ of the retina^18^. During transforming the wide-field image of the spherical eye into the 2-D image, small lesions may be inconspicuous due to distortion^18^. Furthermore, eyelashes and eyelids cover the superior and inferior periphery of the retina in some cases^32^. Aiello et al.^33^ demonstrated that the ETDRS 7SF photography and corresponding fields in the UWF photography have moderate to substantial agreements for DR severity evaluation.

In this study, we configured a deep learning system for DR detection using the ETDRS 7SF image extracted from the UWF fundus image. Although the UWF imaging provides a wide captured area, the far periphery of the retina in UWF images may contain eyelids and eyelashes. Furthermore, to our knowledge, most of the existing deep learning systems for DR detection and evaluation adopt conventional single-field fundus photography. Hence, we extracted and utilized the ETDRS 7SF from UWF images for the DR detection task. By segmenting the ETDRS 7SF from UWF photography, we can save the time and effort for capturing the ETDRS 7SF photography using a single-field fundus camera. To demonstrate the effectiveness of the automated DR detection system based on the ETDRS 7SF images segmented from the UWF photography, we compared the DR detection performance of our system with a system based on the ETDRS F1–F2 images.

From ten runs of ten-fold stratified cross-validation tests with a single run of ten-fold validation, our DR detection system based on the ETDRS 7SF images extracted from the UWF photography achieved a sensitivity of $83.38\pm 0.48\%$, a specificity of $83.41\pm 0.42\%$, an accuracy of $83.38\pm 0.47\%$, and an AUC of $91.50\pm 0.48\%$. For the DR detection system based on ETDRS F1–F2 images, we obtained a sensitivity of $80.60\pm 0.53\%$, a specificity of $80.61\pm 0.69\%$, an accuracy of $80.60\pm 0.54\%$, and an AUC of $88.67\pm 0.37\%$. For all adopted performance metrics, the DR detection based on the ETDRS 7SF images showed around $3\%$ performance advancement over that based on the ETDRS F1–F2 images. Furthermore, we demonstrated that the performance gaps for all adopted metrics are statistically significant via a paired-sample *t* test. As shown in Fig. 8, lesions at the mid-periphery of the retina contributed to the DR detection, where the region is not available in the ETDRS F1–F2 images.

One of the limitations of our approach is that we set an ROI for the DR detection to the ETDRS 7SF among the entire captured area of the retina in the UWF photography. It is to align the image and reduce the influence of obstacles such as eyelids and eyelashes. Automated segmentation of the visible retinal surface without obstructions can be a solution for the limitation. Our immediate future works are automatic segmentation of a larger retinal surface including mid- and far periphery of the retina from the UWF photography and development of the DR evaluation system based on it. Additionally, the data acquired in our study is recognized as single-center, single-ethnicity, and single-device one. For a thorough investigation, the acquisition of multi-center, multi-ethnicity, and multi-device data is essential. Collecting and exploiting such data is one of our future works. Lastly, our system includes the optic disc and macula detection stage, which is indispensable for ETDRS 7SF segmentation. Since the ETDRS 7SF segmentation highly relies on the previous landmarks detection results, failure in the optic disc and macula detection stage results in subsequent unavailability of the DR detection. We deem it as a limitation of our system, where the inclusion of the less restricted image preprocessing is necessary. Probably, a whole-image based DR detection with little segmentation task can be a desirable system.

## Data Availability

The ultra-wide-field fundus image dataset utilized for training, validation, and test was acquired from Catholic Kwandong University International St. Mary’s Hospital, South Korea. This dataset is not publicly available, and restrictions apply to their use. The refuge dataset may be requested from https://refuge.grand-challenge.org/REFUGE2018/.

## Code availability

The code for preprocessing and learning includes intellectual property and cannot be released publicly. However, the ResNet and U-Net algorithms may be acquired from https://github.com/pytorch/vision/blob/master/torchvision/models/resnet.py and https://github.com/usuyama/pytorch-unet. The preprocessing and learning algorithms can be replicated using the information provided in the “Methods” section.
